# The veterinarian as educator: Experiences undertaking an anatomy education extra mural studies placement

**DOI:** 10.1002/ase.70212

**Published:** 2026-03-29

**Authors:** Renato L. Previdelli, Emma Driver, Jane Tomlin, Sarah B. Channon

**Affiliations:** ^1^ Department of Comparative Biomedical Sciences Royal Veterinary College London UK; ^2^ Department of Clinical Science and Services Royal Veterinary College London UK

**Keywords:** employability, extra mural studies, peer teaching, professionalism, veterinary anatomy, workplace learning

## Abstract

Educating clients and teaching and mentoring colleagues are crucial yet underappreciated elements of a veterinarian's professional duties. Unfortunately, veterinary curricula rarely explicitly aim to encourage students to develop effective teaching practices. This study describes a novel 1‐ to 2‐week immersive anatomy education extramural studies (EMS) placement, designed to allow fourth‐ and fifth‐year veterinary medicine students to experience teaching within a professional environment. This study aimed to evaluate the experiences of undergraduate veterinary medicine students and support staff engaged in this opportunity, with a view to exploring the holistic benefits of an anatomy education professional work placement. During 2023–24, 8 students and 4 members of support staff took part in semi‐structured online interviews to explore their placement experiences. Thematic analysis using a grounded theory approach resulted in four themes: Quality Learning, Professional Development, A Safe Space for Personal Development, and The Vet as Teacher. Students were engaged in self‐directed, active and experiential learning, reported enhanced intrinsic motivation for learning, and improved appreciation of the clinical relevance of anatomy. Students developed professional skills, including multiple aspects of communication, and enhanced awareness of diversity. The placement allowed students to develop personal attributes such as self‐efficacy, intellectual humility and uncertainty tolerance in a supportive, safe space. Students strongly identified with the role of a veterinarian as an educator. Consideration should be given to creating formal supported opportunities for students to be actively involved in educating others to enhance student awareness of this crucial professional responsibility and support the development of those necessary skills.

## INTRODUCTION

The importance of developing professional skills and attributes in veterinary students is becoming well appreciated.^1^ In recent years, there has been a growing acknowledgment of the importance of explicitly developing nontechnical skills such as effective communication and teamwork within the curriculum. Historically, such professional competencies have been developed through a reliance on implicit or hidden curriculum messaging through, for example, role modeling.^2^ While the importance of some facets of the veterinary professional's role is well recognized, the role of teaching remains a vital yet commonly overlooked component of a veterinarian's responsibilities.^3^ Veterinarians are frequently required to employ teaching skills when training and supporting colleagues, mentoring placement students within the practice, and educating clients.

A lack of formal training in how to teach is mirrored in undergraduate medical programs.^4^, ^5^, ^6^, ^7^ Several motivations for teaching pedagogical skills to medical students have been suggested, including the future teaching role that medical students have as interns, residents and faculty members; the development of effective communication skills to support interaction with patients; and the assumption that medical students with a better understanding of teaching strategies may become better learners themselves.^8^ Thus, there is a good rationale for increasing the integration of formal teaching opportunities within undergraduate medical and veterinary programs.

Near‐peer teaching is becoming widespread within clinical programs of study, being recognized as an effective approach that enhances both the learning experience and educational outcomes of learners.^9^ Peer interactions can foster a supportive educational atmosphere where students feel more comfortable asking questions and seeking assistance, ultimately enhancing their engagement and motivation in the subject matter.^10^ Peer teaching initiatives have also been shown to facilitate a collaborative learning environment that promotes deeper understanding and retention of complex knowledge.^11^, ^12^ The reciprocal learning process can enhance pedagogical skills and reinforce clinical knowledge, ultimately benefiting the entire educational community.^13^

Peer teaching is considered to enhance the educational experience for students receiving teaching and also contributes to the cognitive and professional development of those who engage in it.^14^, ^15^ As students assume teaching roles, they cultivate essential competencies such as communication, leadership, and teamwork.^16^, ^17^ These skills are particularly pertinent in the veterinary field, where collaboration and effective communication are vital for success in clinical settings.^18^, ^19^ Additionally, teaching peers encourages students to reflect on their own understanding and approaches to learning, thereby enhancing metacognitive skills.^20^ The dual benefits of reinforcing knowledge and developing professional skills position peer teaching as a useful tool, well‐suited to the broader goals of veterinary and medical training to produce competent and collaborative practitioners.

Given its multitude of benefits, the intentional inclusion of peer teaching within medical curricula—rather than as an *“add on”* or optional activity—is increasing, and the positive benefits of this approach are becoming more widely identified.^15^ One key opportunity afforded by this formalization is the potential for a more explicit development of teaching skills to be embedded within the curriculum. Few studies however, have explored how near‐peer teaching specifically contributes to the professional development and teaching skills of peer teachers, with most existing studies utilizing self‐evaluation of peer‐teacher's skills only, through Likert scale surveys.^21^ Thus, there is scope for more qualitative exploration of the experiences of peer teachers in order to understand the process of personal and professional development during near‐peer teaching experiences.

Peer teaching schemes described in the literature are often short‐term and context‐specific. Many existing evaluations of peer teaching and the associated benefits to peer learners and peer teachers, focus on specific schemes within a particular module or subject area (e.g., Refs. [22, 23]), or within a particular domain or setting (e.g., Refs. [24, 25]), or support the implementation of a particular resource or technology (e.g., Refs. [26, 27]). Border et al.^28^ describe the benefits of longer‐term and sustainable near‐peer teaching programs, allowing students to undertake repeated teaching delivery, thus reflecting on and improving their practice. Peer teaching within an immersive workplace learning environment could provide a sustainable model. In this context, near‐peer teachers contribute as part of a wider teaching team across multiple courses, subjects and teaching modes, providing longer term and more integrated opportunities for skill development.

Workplace learning is an essential and fundamental component in the training and development of medical and veterinary professionals,^29^, ^30^ allowing students the opportunity to learn in an authentic, real‐life work environment as they transition into the profession.^31^, ^32^ In the United Kingdom, the Royal College of Veterinary Surgeons (RCVS) requires veterinary undergraduate students to complete Extra Mural Studies (EMS) placements as a mandatory requirement prior to graduation in order to gain practical, real‐world experience in both clinical and professional veterinary settings.^33^

While providing exposure to clinical cases, species, and diverse working environments, EMS placements are particularly valuable in fostering the development of key clinical^34^ and professional competencies, including clinical reasoning, communication skills, and the ability to work within a team.^35^ Given the value of workplace learning for enhancing clinical and professional skills, it follows that workplace learning within a teaching environment may provide students with an opportunity to develop their teaching skills alongside other professional attributes. School teachers' training in the workplace involves three types of learning opportunities: they learn from their teaching experience (for example, through reflection on daily actions in the classroom); from and with others (through interacting and collaborating with other staff, mentors, and experts); as well as from non‐interpersonal sources (through written and digital media, such as books, websites, and social media).^36^ Bringing peer teaching, with its well‐established benefits, into the workplace learning environment may further enhance the benefits of these experiences for students.

This study aimed to develop and evaluate an Anatomy Education workplace learning (EMS) placement. The aim of the placement was for fourth‐ and fifth‐year Bachelor of Veterinary Medicine (BVetMed) students to participate in an immersive peer teaching workplace learning experience within a preclinical academic setting at the Royal Veterinary College (RVC), UK. The primary goal of this study was to investigate the extent to which an EMS placement in Anatomy Education facilitated the development of fundamental competencies essential for veterinary practice. Additionally, the study aimed to identify the broader benefits of an Anatomy Education EMS placement for both placement students as well as staff.

## MATERIALS AND METHODS

Research and experimental procedures involving human subjects in this project received approval from the Social Science Research Ethical Review Board at the Royal Veterinary College (URNSR2023‐0133).

### Curriculum context

A structured workplace‐based learning opportunity in Anatomy Education, lasting a minimum of one week and a maximum of two weeks, was designed and implemented for students in the BVetMed program at the RVC. The BVetMed is a 5‐year integrated program leading to direct registration as a veterinary surgeon. It is organized through body system “strands,” with a spiral structure such that each topic is introduced and then revisited in greater depth and clinical context throughout the program. The final 2 years of the program have minimal didactic teaching, with “clinical” students completing a variety of internally facilitated “core” and “tracking” rotations across different species within the RVC first opinion and referral veterinary environments. During this time, key clinical competencies are developed and assessed. By the end of this phase, students must have completed a minimum of 26 weeks of EMS placements in addition to meeting the requirements of the RCVS, the governing body to which all practicing UK Veterinary Surgeons must register.

EMS placements are elective, that is, chosen by the individual student, and typically take place outside of the veterinary teaching institution. Most commonly, students will undertake these placements at a number of different veterinary hospitals/practices, however it is possible for students to elect to undertake a limited amount of “professional EMS” to allow them to explore various alternative career opportunities outside of clinical practice. EMS placements in the United Kingdom share similarities with the clinical electives required in the fourth year of the Doctor of Veterinary Medicine (DVM) curriculum in North America, as both are opportunities in which students have the autonomy to choose where to complete them, tailoring their learning experiences to their individual interests and career goals.

The Anatomy Education placement was designed to provide students with a professional EMS opportunity within an educational setting, to showcase alternative career opportunities for students who may be considering careers outside of veterinary medical practice or academia. Further aims of the placement were to facilitate collaboration and educational opportunities between clinical rotation students and preclinical students, who would not normally interact due to the geographical separation of the two RVC campuses, as well as to increase collaboration and interactions between preclinical staff and students.

### Anatomy Education EMS placement

The anatomy education EMS placement was offered as a 1‐ to 2‐week professional EMS placement opportunity, hosted by the anatomy teaching team within the Department of Comparative Biomedical Sciences (CBS) at the RVC, during teaching term time. All 4th‐ and 5th‐year BVetMed students were eligible to sign up to attend the EMS placement on a voluntary basis; however, a maximum of two EMS students were accepted onto the placement at any one time, and the placement was offered only during term time. The placement was fully subscribed and places were booked on a first‐come, first‐served basis.

Prior to starting the placement, students were provided with bespoke information, including a timetable detailing the teaching sessions they were to attend and further goals or tasks that they were expected to achieve while in attendance, such as anatomical specimen preparation or teaching resource development. EMS students were allocated a member of the anatomy teaching staff as a point of contact and mentor. A mandatory online health and safety induction was completed at the start of the placement. A selection of curated online resources was also provided at the outset of the placement to support EMS students in developing foundational pedagogical knowledge and techniques to apply during their interactions with first‐ and second‐year BVetMed students learning anatomy.

On the last day of the placement, EMS students undertook a feedback discussion with their mentor and completed a reflective written portfolio entry about their placement experience. The mentor also completed and submitted a formal feedback assessment of each EMS student's performance to the RVC EMS office to confirm the completion of this EMS placement.

### Data collection

This research received ethical approval from the Social Science Research Ethical Review Board at the Royal Veterinary College: URN SR2023‐0133 before any participant was contacted and data collected. All participants read the participant information and provided written consent before engaging in this research.

EMS students in the academic year of 2023–24 who agreed to participate in the study (25 of 27 students) received a short questionnaire to complete before attending their placement, establishing their reasons for undertaking the placement, their expectations, and demographic information, including whether students had any previous experience of teaching. Upon completion of their EMS placements in Anatomy Education, the 25 participating EMS students were invited via email to participate in a semi‐structured online interview to review their experience and discuss their perceptions of their EMS placement. Eight students agreed to take part in interviews. Only selected staff were invited to participate, based on achieving a balanced view across various staff roles.

Interviews were conducted using Microsoft (MS) Teams® software with 8 EMS students (one 4th Year BVetMed student and seven 5th Year BVetMed students) who consented to participate in this research study. Interviews were facilitated by a staff member who was not directly associated with the placement (ED), ensuring an unbiased approach to data gathering. Participants were asked open‐ended questions about their placement experiences, which allowed in‐depth discussions, enabling participants to reflect on their experiences and articulate the perceived benefits and challenges of the EMS placement. The purpose of the interviews aligned with the study aims which was to explore the experiences that participants had on placement, the benefits (and required improvements) of the placement, and to understand how the placements assisted with the development of fundamental competencies essential for veterinary practice. The interview guide is provided in Supporting Information Appendix 1. By using a virtual platform, researchers were able to overcome geographical barriers, as students were still engaged in the clinical workplace‐based learning part of their curriculum at the time of the study. Following the completion of the student interviews, four staff members supporting the placements (one member of the technical team, two teaching fellows of veterinary anatomy, and an associate professor of veterinary anatomy) also participated in interviews using a modified version of the interview questions. Staff interviews aimed to capture the benefits of the placement to students from the staff perspective, as well as investigate the benefits and challenges of hosting the placement. Staff were selected to obtain a range of role types (teaching versus support) and seniority, and to avoid interviewing staff involved in the design or administration of the placement.

### Data analysis

Qualitative preplacement survey data underwent content analysis to categorize student preplacement expectations. Interview data were transcribed using the transcription function within MS Teams, checked, and anonymized before data analysis took place. Qualitative analysis was conducted, employing reflexive thematic analysis through a grounded theory approach with a critical realist positionality. Following familiarization with the data, the data were manually coded on hard copy printouts of transcripts and through an iterative process of identifying themes, revisiting and revising codes and themes, clearly refined and defined themes were developed. Grounded theory applied in this study enabled researchers to derive theories directly from the data, rather than imposing pre‐existing frameworks (Holton).^37^ By grounding findings in the actual experiences of the participants, the methodology ensured that the conclusions drawn were both relevant and reflective of the complexities inherent in the peer teaching experience within the context of anatomy and veterinary education.

### Reflexivity statement

As education‐focused academics, our backgrounds and interests may have influenced the course of this research. The four authors of this study were all active researchers in the evaluation of the EMS placement experience. Coupled with this, two authors (SBC and JT) were the original creators of the anatomy education EMS placement, while at the time of data collection, RP was involved in administering the placement and supporting all students who were undertaking the placement. SC was involved in anatomy teaching during the study and had professional interactions with the placement students throughout the teaching day. Our close involvement in the design, running, and support of the placement students undoubtedly influences the perspective with which the research project was designed and will have shaped the interpretation of the data during analysis. Spending time with the students and observing their development and experience first‐hand makes it impossible to read and analyze interview scripts from an uninformed and impartial viewpoint. Instead, we acknowledge and bring our lived experience of the placements to strengthen and complement the analysis and interpretation of the data.

ED was chosen to undertake the participant interviews, as the only researcher who was not actively involved in the placement at the time of the study. ED's perspective and interest in the placement arises from her prior involvement in administering the placement during previous placement years, and from her interest and curriculum leadership in the area of professionalism. This position allowed ED sufficient insight into the workings of the placement to support the conduct of interviews with the participants. However, her impartiality and lack of direct involvement during the study period allowed participants to provide her with candid and honest responses about the placement in a comfortable environment. As a familiar member of the academic teaching staff of the institution, however, we must acknowledge the potential power dynamic during interviews.

## RESULTS

Of the 27 BVetMed students who participated in the Anatomy Education EMS placement in the 2023–24 academic year, 25 opted to participate in this study, of whom 19 were in the 5th year and 6 were in the 4th year. Eleven participants reported having prior teaching experience preplacement. Participants expressed various expectations for the placement, which included the desire to solidify, refresh, or gain new anatomical knowledge, indicating a clear motivation to enhance their understanding of the subject matter. Additionally, EMS students aimed to help their peers learn, and to exchange ideas and knowledge with staff and other students. Further expectations included a desire to improve communication skills for future interactions with clients and to experience teaching to confirm an existing interest in a teaching‐related career path.

**FIGURE 1:**
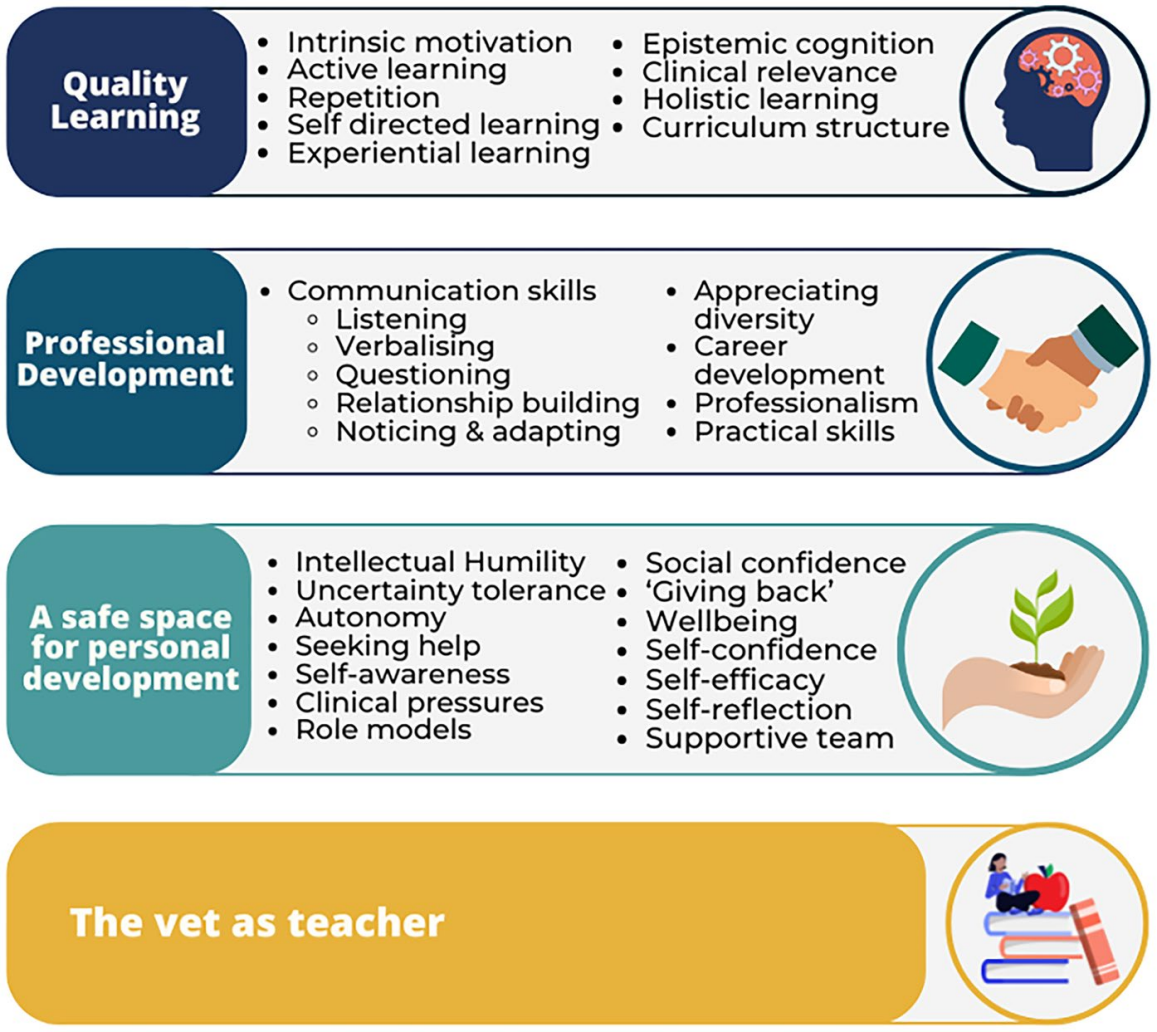
Thematic map indicating the four key themes, and the codes within which led to development of the themes.

Analysis of staff‐ and student‐reported placement experiences resulted in the development of four key themes: Quality Learning; Professional Development; A Safe Space for Personal Development; and The Vet as Teacher (Figure 1; Table 1).

**TABLE 1:** Themes, theme descriptions and example quotes to illustrate the essence of each theme.

Theme	Theme description	Example quote
Quality learning	Students and staff described ways in which learning was high quality, for example, self‐direction, active/experiential approaches and enhanced motivation for learning	*“[*…*] there was a couple of times where I had to be like, ‘oh you know I don't actually know this’, like, ‘let's go find somebody else to, like, walk us through it’. But then once I obviously got that information, I felt better about, if somebody asked me again, I now know what the information was.”* [Student 6]
Professional development	Students developed a range of professional skills on placement, including facets of communication, practical skills, and general professionalism	*“I had to adapt my approach […] there was a variety of kind of personalities that you would be exposed to and because of that you would have to kind of address the question or formulate the question in a slightly different way depending on what kind of student you were working with*…*”*
A Safe Space for Personal Development	The comfortable and safe learning environment supported students to grow personally in a range of areas, for example in the development of non‐cognitive attributes such as self‐efficacy and uncertainty tolerance	*“It was quite nice that people kind of trusted me to like ‘OK you can go answer this’ [*…*] so that made me a little bit, you know, more confident myself and kind of gave me a boost, an ego boost, you know, which was nice.”*
The Vet as Teacher	Students developed their understanding of the importance of teaching skills within the veterinary profession, developing their professional identity through the development of these skills	*“No matter where you go, when you become a veterinarian […] you're going to have to teach something to somebody, no matter how small or large [*…*] it's really important to be able to break things down to somebody and teach them a new concept*…*”*

### Quality learning

Students undertaking the Anatomy Education EMS placement were immersed in opportunities for high‐quality learning. Students reported considerable self‐direction in their learning, coupled with a heightened motivation for learning. Engaging in a teaching role fostered a sense of responsibility for their own educational needs in order to fulfil the expectations and needs of their peers.

I was prepping the night before… I'd have what I found and potential questions that they could ask me written out on them. [Student 1]

The dynamic interactions with staff, students and teaching resources within teaching sessions led learning to be active, authentic, and experiential, with students required to apply their knowledge in a practical context, reinforcing their understanding of anatomical concepts.

They're almost experiencing the session as if they were a student as well. And you can tell they're remembering their previous anatomy teaching but then maybe they're asking us some extra questions to remind them which kind of helps to solidify it. [Staff 1]

I remember doing this 6 years ago and I've learned so much more now about the actual practical due to the fact that I have to be able to answer questions on it. [Student 7]

[…] there was a couple of times where I had to be like, ‘oh you know I don't actually know this’, like, ‘let's go find somebody else to, like, walk us through it’. But then once I obviously got that information, I felt better about, if somebody asked me again, I now know what the information was. [Student 6]

In addition, the repetitive nature of facilitating multiple similar teaching sessions or interactions further solidified student learning.

Especially with some of the sessions where we have done 4 sessions back to back, by the end of that fourth session I knew what I was talking about and everything from the back of the brain had come forward and I was like ‘yeah, we got this now’. [Student 1]

The placement experience fostered student's understanding of the relevance of anatomy to clinical practice, complementing the existing spiral curriculum structure.

After this week I realised how important it is to have like a really good basic knowledge of all the animal anatomy and that […] when I was talking to some of the students I kind of talked about the anatomical stuff, and then I went on to the clinical stuff. And then I realised how important the relationship is between the anatomy and the clinical stuff. [Student 2]

It's coming back with that extra layer of knowledge and then being able to utilise that […] it's another checkpoint in the spiral curriculum of them being able to assess, OK, I've gone away, and I've got this extra knowledge from doing placements […] and actually what's relevant and how it fits together. [Staff 2]

Participating in the placement facilitated holistic learning, integrating various dimensions of knowledge and skills, and developing students' epistemic cognition (thinking about knowledge and knowing): students became more aware of their learning processes and how to navigate complex concepts effectively.

So maybe not even as much as I've learned new things. It was more just simply the fact that I've understood how it all connects. [Student 4]

I was able to definitely incorporate the veterinary knowledge and things that I've already learned into kind of more in‐depth … ‘why is it here, why do we find this useful’? [Student 6]

### Professional development

EMS students engaged in the peer teaching placement reported an impact on their professional development, particularly in areas such as practical skills and communication. Students developed practical skills within the dissection room as well as during specimen preparation and resource development tasks.

Definitely instrument handling, that was a difficult one at the start and actually I've improved quite well with that. [Student 7]

I know they have definitely worked on and built on their dissection, or you might want to call them prosection skills actually, not just dissecting to find something, but dissecting to make it look nice so someone else can identify it as well […] we've taught somehow to actually embalm […] putting the cannulas in, stitching them up afterwards. [Staff 3]

EMS Students and staff reported that multiple facets of communication skills were also developed. Students learned to articulate complex concepts clearly and effectively.

Definitely finding the correct kind of teaching styles and wording that you need to give for the students because you know it works a certain way in your brain, but it's like, how do you apply that knowledge to people who don't know it? [Student 6]

So the week actually gives me a way to try to find out my own way of talking and not to say too much more than I needed to but make sure that you know what I'm trying to tell you. [Student 2]

It's an extremely challenging environment, the teaching environment actually […] there's a huge amount of information and an awful lot of people. And a big mix of academic content, and also necessary professional skills to bring all that together into being an effective communicator. [Staff 4]

EMS students developed active listening skills in order to understand what was required of them, allowing them to respond thoughtfully to peer inquiries and feedback. They also became adept at noticing the diverse needs of their peers, such as their varied backgrounds and perspectives, and adapting their teaching methods and explanations in real‐time accordingly.

Their thought processes were different to mine so I really had to pay attention to what they were saying […] to be able to fully understand their question so that I wouldn't just go on with something irrelevant […] It was really that analysis of what they were saying, to just give them exactly what they want. [Student 4]

I had to adapt my approach […] there was a variety of kind of personalities that you would be exposed to and because of that you would have to kind of address the question or formulate the question in a slightly different way depending on what kind of student you were working with […] I guess the different types of questions and the different types of people that were asking those questions and kind of determining ‘OK, are you going to be OK if I just verbally tell you something or do I have to actually like show you’. [Student 3]

Additionally, this EMS placement in Anatomy Education contributed to the overall career development and professionalism of students, equipping them with competencies that are essential for future success in the veterinary field or beyond.

Like generic professionalism skills of, you know, turning up on time, communicating, the fact that they are treated as a colleague versus a student. [Staff 2]

Because the profession, the retention of people in clinical practice, especially new grads is, is changing, I think it's good for them to see like actually there was another option and not everyone chooses that path. [Staff 1]

### A Safe Space for Personal Development

EMS students identified the placement as a safe space that significantly contributed to their personal development. The experience of “giving back” to their peers fostered a sense of reciprocity, community, and mutual support.

[…] that [providing clinical context] kind of helped me and the student that I was with kind of solidified what we were trying to accomplish together. [Student 3]

Being in this environment, where like it's an education sphere – you're dealing with students and teachers ‐ you feel very comfortable, it's like we're all either in the process of pursuing vet or like you've already gone through that process. So it feels like it's a very safe network to work in. [Student 5]

I love having the EMS students. I think they are massively enriching to our lives as well as to the students that they're helping teach. [Staff 2]

Both students and staff identified the value of the placement in temporarily providing students with a lower pressure environment, far from the demands and high‐stakes environment of clinical rotations, with potential benefits for student well‐being.

I imagine if I was them […] you're going to eat better, sleep better, so you're going to come in maybe with more energy. [Staff 1]

I think when you're talking to your peers its different […] but when its somebody that you know eventually will be a client, they're looking to you for that information and you have to be that figurehead for them that goes and gets it. And so, I definitely think when you're in that position it's a lot different and it's nice to kind of have that experience before you go into a situation where you're like, ‘Oh no, it's just me now, what do I do?’ [Student 6]

EMS students valued the relative autonomy within this placement, which not only fostered independence in their learning but also instilled a sense of being a valued trusted and respected team member. This, coupled with the support from the wider teaching and technical team, empowered students to speak out and ask for help, a trait that some reported had been lost or implicitly discouraged within clinical environments.

I think just the fact that we were allowed to get on with it…and if we needed help obviously the anatomy team were there…but it was pretty much, ‘Yeah, go figure it out, we're here if you need us.’ [Student 7]

I think the team are like, ‘we all will admit that like we don't know everything, we need to ask for help’. So like trying to instil that in them is a good thing to demonstrate. [Staff 1]

Some students reported improved social confidence as they moved past social discomfort, and grew to enjoy interacting with their peers and staff.

I found it really useful in terms of public speaking, talking to people that I don't know […] just getting used to talking to strangers […] I stress about talking to strangers and saying things that I don't mean to, or I get nervous and then things just come out, so we kind of build up the confidence to know I can do it. [Student 2]

The requirement to recall prior learning, coupled with interacting with peers at an earlier stage of learning development, resulted in EMS students reporting increased self‐efficacy and enhanced self‐confidence. Through self‐reflection and supported by trust and positive feedback from staff and their peers, EMS students were led to appreciate the true extent of their knowledge and abilities.

[…] once the nerves are gone, like, ‘oh am I saying the right thing?’[…] you know I do know what I'm talking about! I've come quite a far way from where I was at the beginning, first year to now. [Student 6]

And I was like, ‘I actually know something that I can actually teach to people’. And in one of the dissection sessions […] I was talking to one of the groups and then one of them was like ‘you're actually doing a really good job’ and I was like ‘thank you’. I really needed that! [Student 2]

It was quite nice that people kind of trusted me to like ‘OK you can go answer this’ […] so that made me a little bit, you know, more confident myself and kind of gave me a boost, an ego boost, you know, which was nice. You don't get a lot of those in veterinary medicine. [Student 3]

The placement fostered intellectual humility as EMS students recognized the limitations of their knowledge, as well as those around them, and the importance of lifelong learning.

Other people that I was working with referred to those textbooks all the time and it made me feel kind of a lot happier about the fact that OK, it's OK to actually not know everything on the spot. It's OK to refer back to the things and remind yourself of certain things that you might not have necessarily covered. [Student 3]

It's better than giving them a fake answer for sure. Everybody can't know everything. If there's something that's more complicated I don't want to give them the wrong answer and make their knowledge wrong or hurt their pet. I don't want that to happen. So I'd rather say ‘hey, I don't know, maybe this isn't my expertise, but let me figure it out or give it to somebody who does know this.’ [Student 6]

Navigating these inherent uncertainties within peer teaching encouraged tolerance for ambiguity, preparing students for the complexities of clinical practice.

I don't know any of the clients, I don't know all the questions that they will ask, I don't know all the answers that they want. And it gives me an insight into how to communicate with people that I don't know about things that I don't know. Like, I can't just say ‘I don't know, sorry’. But I now found… the mindset of how even though I don't know the answer to the question that you're asking me, I can go away, look it up and get back to you. [Student 2]

### The Vet as Teacher

Through their engagement in peer teaching in anatomy, students developed a nuanced understanding of the imperative role that teaching will play in their future careers as veterinary surgeons. This experience highlighted the necessity of effectively conveying complex information, not only to peers but also to colleagues and clients, reinforcing the idea that veterinarians must serve as educators in various contexts. By articulating anatomical concepts to fellow students, they recognized the importance of clear communication in fostering comprehension and trust, skills that are essential when interacting with staff and clients who may lack specialized knowledge.

No matter where you go, when you become a veterinarian […] you're going to have to teach something to somebody, no matter how small or large […] it's really important to be able to break things down to somebody and teach them a new concept. And the ability to do that is, I guess, undervalued […] I think if more people were comfortable with the concept of teaching it would improve the profession and improve morale, communication between teams and all that kind of stuff. [Student 3]

At some point whichever path you go down, you will have to teach, whether that's your colleague, older or younger than you, nurses and to like an extent you teach the client about a lot of things as well. [Staff 1]

EMS students related their growing appreciation for the diverse backgrounds and learning needs of their peers to the challenges they would likely face in clinical practice. They demonstrated an understanding that adaptable communication strategies were fundamental in establishing rapport and building relationships in professional practice, essential for successful client interactions and teamwork within veterinary settings.

If you're in general practice you know people are bringing you their pets and they don't really know what's wrong and you have to communicate with them […] but they're not at this level […] if it's something that's simple, if it's more complex, you still have to communicate the issues […] people look to you for that information and that knowledge. [Student 6]

Ultimately, this peer teaching experience not only enriched students' anatomical knowledge and developed practical skills but also prepared them for the collaborative and educational responsibilities they will encounter as future veterinary professionals.

## DISCUSSION

This study aimed to implement and evaluate a near‐peer anatomy education workplace learning experience within an undergraduate veterinary curriculum, with a focus on the development of professional competencies in the near‐peer teachers. The results identify several areas through which professional competence was enhanced by completing this placement: knowledge development, professional skills development (including practical and communication skills), an enhanced understanding of the role of the veterinary professional, as well as the development of a range of personal attributes. Critically, two of the major themes, Quality Learning and A Safe Space for Personal Development, allow us to understand the nuances underpinning how the development of enhanced competencies was supported through the placement activities and environment.

### Learning through experience

Student perspectives on their anatomy education EMS placements emphasize the significant role of this experience in enhancing their learning. They described quality, dynamic interactions with staff, fellow students, and teaching resources, and reported that their learning was active, self‐directed, authentic, and experiential, as they were required to apply their theoretical knowledge in practical, real‐world contexts.

Student experiences align closely with the principles of experiential learning theory.^38^, ^39^ All four stages of Kolb's experiential learning theory can be identified within the experiences of Anatomy Education EMS students as they first encounter anatomical information during the early years of the veterinary program (concrete experience), reflect on their knowledge through the lens of their clinical experience when preparing for their peer teaching sessions (reflective observation), integrate this learning into their existing understanding of the topic or the profession (abstract conceptualization), and then seek to apply this new knowledge in future teaching and learning interactions (active experimentation).^39^ It is likely that engagement in such reflective processes underpins the reported changes in epistemic confidence (the confidence in being correct in your own knowledge and conclusions) by students, which links strongly with the skill of metacognition and the critical professional competency of being aware of one's own limitations.

While the personal development of students aligns well with Kolb's model, the considerable emphasis placed by students on the strength of the placement environment and social interactions aligns most clearly with Dewey's experiential learning theory in that the learning community and the social context of education are paramount in the construction of knowledge.^38^ During the Anatomy Education EMS placements, students interacted with a variety of individuals: peers, technical, and teaching staff from a range of backgrounds and specialties. This social interaction fostered a collective learning process in which students exchanged ideas, engaged in problem‐solving discussions, and shared diverse perspectives that enriched their understanding of anatomy. This approach reflects Dewey's belief that learning is inherently relational, with the social context serving as a vital component in shaping and consolidating knowledge. It also aligns with recent findings that near‐peer teaching within the clinical workplace environment promotes the learning of peer teachers through the development of a community of practice and through reflective practice.^40^ It is notable that the current initiative took place outside of the clinical environment, suggesting that perhaps the key feature driving the learning process is the workplace and community of practice itself and not the specific clinical teaching matter.

### The Anatomy Education EMS Community of Practice (CoP)

The Community of Practice^41^ developed at the inception of the Anatomy Education EMS teaching placement appears to be fundamental to the skills, attributes and knowledge developed by placement students. According to Wenger,^41^ a community of practice is characterized by three essential components: the domain (the shared interest, which in this case is veterinary anatomy), the community (the group of people for whom the domain is relevant, in this case, all peer‐teachers, preclinical students, as well as the staff supporting these two categories of learners), and the practice (the body of knowledge and resources shared within the community, which in this case includes the knowledge, behaviors, and teaching methods held by the group). It is likely that the embodiment of the “safe space for learning” reported by placement students, lies in the effective community of practice, where sharing of resources (i.e., through effective role modeling), joint enterprise and mutual engagement support the development of not only knowledge and skills, but also professional attitudes and behaviors. A community of practice facilitates legitimate peripheral participation, where newcomers gradually move toward full participation in a community by learning to navigate their roles effectively.^41^ This description well mirrors the experiences of Anatomy Education EMS students.

Students reported changes in personal attributes and attitudes that are considered valuable in medical or veterinary professional competence, and often linked the development of these traits to staff modeling of effective behaviors. One example is intellectual humility—the awareness and acknowledgement of one's own intellectual limitations and others' intellectual strengths.^42^ The behavioral traits of an intellectually humble individual include admitting ignorance and mistakes and seeking help to learn.^43^ Many who teach veterinary and medical professionals will recognize these as important actions in the clinical workplace, but in this study, educators were also seen to effectively demonstrate such behaviors, promoting improvements in this trait in placement students. Similarly, students reported that initially they found ambiguous or unclear situations to be challenging, but that the placement promoted their tolerance of uncertainty. This aligns with previous studies that indicate that the anatomy learning environment is bountiful in opportunities to support learners with developing this trait.^44^ Notably, research in this field also emphasizes the educator's role in modeling acknowledgement of uncertainty^45^ and helping students to engage in the process of reflective learning.^46^ This further indicates the strong influence of an effective community of practice while undertaking an anatomy EMS placement.

### Personal development within the CoP

Safe spaces are vital in allowing learners to navigate the complexities of their developing professional identities.^47^ A learning environment that facilitates vulnerability and challenge, inherent to learning and growing in a demanding field, ultimately enables students to develop noncognitive attributes that will serve them well in their future careers.^48^ Students described significant personal development while on placement, notably in self‐confidence and self‐efficacy. Such traits are essential foundational elements for students' emerging professional identities as veterinarians.^31^

Bandura^49^ suggested that a person's belief in their own efficacy is developed by four types of influence: mastery experiences, vicarious experiences, social persuasion, and emotional states. Students frequently reported mastery experiences, describing the value of teaching multiple repeats of teaching sessions, their deliberate self‐directed preparation, and their resulting pleasure in feeling competent and able to teach the subject matter. Peer teachers frequently compared their own performance with their peers, reflecting on and acknowledging the progress they had made during their time in their program (vicarious experiences). Students were influenced by the feedback they received from their peers and staff (social persuasion) and likely by their (positive) emotional state. Maddux^50^ added a fifth influence on self‐efficacy: imaging/visualization, relating to a person's ability to see themselves being effective in a given situation. The theme “Vet as Teacher” demonstrates that, through this placement, students are starting to draw parallels between their teaching interactions within the classroom and their future interactions with clients in clinical settings. Thus, the reported increases in self‐efficacy during this placement likely come via multiple spheres of influence.

Improvements in self‐efficacy and the influence of mastery experiences within this placement align with the “Competence” aspect of Self Determination Theory of motivation (SDT).^51^ Ten Cate and Durning^15^ have previously identified the benefits of near‐peer teaching as being well supported by SDT, with intrinsic motivation of peer teachers noted to be high. The intrinsic motivation of peer teachers (see Quality Learning theme) and the other two SDT components that support this intrinsic motivation—autonomy and relatedness—are well evidenced within the current study. Students highly valued the relative autonomy afforded to them during the anatomy education EMS placement, while their reported sense of belonging, in being part of a supportive team, may contribute to a sense of relatedness. A core condition for clinical workplace learning is “supported participation”,^52^ and it follows that enhanced relatedness may underpin the anatomy education EMS placement environment being attributed as a “safe space” by students undertaking this placement. This seems likely, given the importance of communities of practice for the learning of peer teaching residents within the clinical workplace.^40^

### Professional competencies and the professional identity

Peer teachers developed a range of practical and professional skills while undertaking an anatomy education EMS placement. Multiple facets of communication were developed: general communication skills and the ability to verbalize explanations were frequently noted, but also more nuanced aspects such as listening, noticing, and questioning, as well as communicating with diverse individuals were learned and enhanced during this placement. The relevance of such key skills to the role of a veterinary professional, especially (but not exclusively) when interacting with clients in a clinical consultation, is clear.^18^ While the current study was not conducted in a clinical environment, the associated development of practical clinical reasoning skills, it is clear that EMS conducted in a professional environment has the potential to develop relevant skills that are transferrable and applicable to the clinical context.

The development of practical skills in the context of veterinary anatomy provides a mechanism through which final‐year veterinary students can begin to enact elements of their future professional roles. According to Cruess et al.,^47^ professional identity formation arises through the internalization of professional values and behaviors, a process facilitated by authentic practice and social participation within a community of learning. Within the EMS teaching environment, these experiences not only reinforce technical proficiency but also cultivate the professional dispositions necessary for the transition from student to practitioner.

### Limitations and outlook

Our findings, derived from a small sample of 8 EMS students from a single institution, have limitations in terms of their generalizability to broader contexts. The specificity of the sample (exclusively veterinary students and from only two cohorts) may narrow the applicability of the findings. How these might transfer to other healthcare professions' curricula is unknown. Educational strategies, such as our EMS initiative, which promote experiential learning and communication skills, are vital across various healthcare disciplines, yet contextual factors unique to veterinary education may influence outcomes differently in other fields. Similarly, diverse learner populations might impact how professional competencies and personal attributes develop during such initiatives.^53^ Furthermore, diverse learning environments and cultures across different higher education institutions, and varied professional challenges faced by different health‐related professionals, mean that not all experiences on such a program may align directly with those of veterinary cohorts. Nevertheless, our study contributes valuable initial insights and further research evaluating such educational initiatives within other settings would support further exploration of the potential similarities and differences across disciplines and contexts.

Students elected to participate in the EMS placement (opt in). Their reasons for doing so varied, with some students suggesting they had an intrinsic interest in teaching, some recalled positive peer teaching interactions in their learning journey, and some recognized that teaching experience might be beneficial in future applications to academic veterinary posts such as internships and residencies. Some students were not attracted by the teaching; moreover, the exposure to basic sciences ahead of preparation for finals and other licensing examinations. Nevertheless, students who volunteered for this placement knew that the placement expected them to teach. It is likely, therefore, that this unique EMS placement, dedicated to peer teaching, attracted those students who are more comfortable with the type of interpersonal interactions that teaching requires. There are clear benefits to this: the enthusiasm and commitment of students who chose this placement significantly contributed to an enriching learning atmosphere for all junior and senior students. However, an aspirational goal of the EMS placement would be for all students, especially those who place less value on teaching skills as an essential professional competency, to be attracted to complete this placement. Given the importance of the community of practice to the development of placement participants, caution should be applied when considering making any such scheme or initiative mandatory, as reluctant participants may impact the community dynamic. Anecdotally, we have found that through running this initiative, we have seen a cultural shift where preclinical students are influenced to participate in the placement through their direct exposure to the EMS students. A future aim of our research is to evaluate the longer‐term impact of the placement, as well as its impact on the students being taught by placement students. Longer term, consideration of how some of the benefits of this placement could be delivered within the formal “core” curriculum would potentially expand the reach of this work to those students who do not elect the optional placement, or who cannot participate due to oversubscription.

This study introduces and evaluates an initiative in a single curriculum and institution—a UK small specialist veterinary school with an integrative systems‐based spiral curriculum—and so is vulnerable to context‐specificity. Despite this, our findings align with many of those from previous studies of near‐peer teaching, adding to our understanding of how such initiatives can be optimized for maximum learning benefits. In a similar vein, our methodology is qualitative, which lacks the repeatability and generalizability of results when compared to quantitative or mixed methods approaches. The strength of our qualitative approach, however, lies in the rich depth of understanding of the individual experiences of near‐peer teachers and staff, allowing us to appreciate not simply the presence or magnitude of learning gains, but instead, the nuances of personal experience that underpin why and how this and similar initiatives are of value. It would, of course, be ideal to supplement the data presented in this study with further quantitative data to allow us to measure the development of skills such as professional and teaching skills, and this will be the focus of future work. Overall, our results present the value of comprehensive workplace‐centered peer teaching experiences in veterinary education and indicate that the benefits associated with near‐peer teaching extend beyond isolated sessions, contributing significantly to the holistic development of students. Future research should continue in order to explore these experiences to further elucidate the potential for enhanced learning outcomes in different areas of the veterinary curriculum.

### Practical recommendations

The integration of Anatomy Education workplace learning opportunities into a curriculum presents a unique opportunity to enrich learning experiences and promote professional development. Here, we provide recommendations to support other anatomy educators in implementing similar teaching‐focused workplace‐based peer teaching experiences:

- Immersive peer teaching experiences are valuable: Consider how you might provide space for an immersive peer teaching opportunity—perhaps this could be during a vacation (for example, aligned with a summer school), as an elective experience, a module in its own right, or could fulfill work experience requirements. Spending time immersed fully within a teaching team brings additional benefits to those arising from regular peer teaching initiatives.
- Structured learning objectives: Define clear learning objectives that outline the desired outcomes of both teaching and learning aspects of the EMS placement, such as improved communication skills and enhanced understanding of the curriculum.
- Role model effective and professional practices: Faculty, support and technical staff should understand the goals of the placement and should strive to serve as role models, actively demonstrating and modeling effective teaching practices for students to observe and replicate. Modeling uncertainty as a normal professional encounter and sharing strategies to approach uncertainty, can foster intellectual humility and tolerance of ambiguity in peer teaching students.
- Provide mentorship, guidance, and feedback: Assigning a mentor, or key contact who can support students during their teaching placements, allows for the provision of continual feedback, dialogue, and promotes professional interactions.
- Provide pedagogical training for participants: provide reading materials and structured learning opportunities (e.g., journal clubs) to support placement students in understanding the underpinning teaching and learning theory
- Establish safe spaces for learning: Promote an environment that fosters collaboration and mutual support among staff and students (e.g., efficient communication, friendliness, and respectful attitudes).
- Build in repetition, and nonteaching time: Where possible, require students to teach the same session multiple times, either to different cohorts or to multiple subsets of a cohort—the repetition is valuable to allow them to build confidence and learn from mistakes. Nonteaching time is essential to allow peer‐teachers to prepare prior to sessions and reflect subsequently.
- Encourage reflection in learning: Model reflective practices where students consider their teaching experiences, how they might build on these, and how these relate to their future roles as veterinarians, thereby reinforcing the importance of professional identity formation as discussed in Cruess et al.'s.^47^

## CONCLUSION

An Anatomy Education workplace learning opportunity promotes quality learning of anatomy, and the development of veterinary professional competencies by developing multiple professional skills and personal attributes in a supportive, safe environment. The benefits of this initiative extend beyond the student peer teachers to include teaching staff and the institution as a whole as well as the students being taught by peer educators.

The insights gained from this research provide a foundation for future developments in peer teaching initiatives, highlighting the need for diverse evaluation approaches and ongoing adaptation to ensure that these programs continue to meet the needs of both students and faculty. We share recommendations to support anatomy educators who wish to develop similar experiences within their own settings.

## AUTHOR CONTRIBUTIONS

**Renato L. Previdelli:** Conceptualization; investigation; writing – original draft; methodology; validation; visualization; writing – review and editing; project administration; formal analysis; data curation; resources. **Emma Driver:** Investigation; conceptualization; validation; writing – review and editing; formal analysis; data curation; software. **Jane Tomlin:** Writing – review and editing; visualization; validation. **Sarah B. Channon:** Project administration; conceptualization; investigation; funding acquisition; methodology; validation; visualization; writing – review and editing; supervision; data curation; formal analysis.

## ETHICS STATEMENT

Ethics approval by the Social Science Research Ethical Review Board at the Royal Veterinary College: URN SR2023‐0133.

## Supporting information


**Appendix S1:** Supporting Information.
