# Supplementary material for: The veterinarian as educator: Experiences undertaking an anatomy education extra mural studies placement
Source: Anat Sci Educ. 2026 Mar 29;19(5):709–22. doi: 10.1002/ase.70212 (PMC13184585; doi:10.1002/ase.70212)
Supplement: Supplementary file 1 — Appendix S1: Supporting Information. [file ASE-19-709-s001.docx]

Appendix 1: **Semi-structured interview guide:**

1. **What was your reasoning behind choosing an EMS placement?**

**Remind participant of their pre-placement expectations**

1. **Do you believe your expectations were met upon completion of this EMS placement?**
2. **Were they met or not?**
3. **If not, what could’ve happened differently to meet them?**

1. **What skills have you developed on this placement?**
2. **Can you share an example of when it was used or developed during your placement?**

1. **How do you think the EMS placement has developed your anatomy knowledge – can you give some examples?**

1. **What elements of this EMS Placement in Anatomy Education do you think will help you the most in your future career as a vet? And why?**

1. **How important is it for you to have a formal teaching experience opportunity (like this EMS placement) during your studies?**
